# miR-543 regulates high glucose-induced fibrosis and autophagy in diabetic nephropathy by targeting TSPAN8

**DOI:** 10.1186/s12882-022-02716-8

**Published:** 2022-03-04

**Authors:** Langen Zhuang, Xiaoxu Ge, Xiaolei Hu, Qingqing Yang, Xiaoyan Pei, Guoxi Jin

**Affiliations:** 1 Department of Endocrinology, The First Affiliated Hospital of Bengbu Medical College, 287 Changhuai Road, Longzi lake District, Bengbu, 233004 Anhui Province China; 2 Department of Endocrinology, Tongren Hospital Affiliated to Jiaotong University, Shanghai, China

**Keywords:** Diabetic nephropathy (DN), miR-543, TSPAN8, fibrosis, autophagy

## Abstract

**Background:**

Diabetic nephropathy (DN) is one of the most common and serious complications of diabetes, which can lead to renal failure and fatality. miRNAs are an important class of endogenous non-coding RNAs implicated in a wide range of biological processes and pathological conditions. This study aims to investigate the potential functional roles of miR-543 in DN and its underlying mechanisms.

**Methods:**

qRT-PCR was performed to detect the expression levels of miR-543 and TSPAN8 in kidney tissues of mice with DN. Western blot (WB) was used to measure the protein levels. CCK8 assay was employed to evaluate the proliferation of HK2 cells. Dual luciferase reporter assay was conducted to verify the functional interaction between miR-543 and TSpan8.

**Results:**

The downregulation of miR-543 and upregulation of TSPAN8 were observed in kidney tissues of mice with DN. miR-543 mimic significantly decreased cell proliferation and autophagy in high-glucose (HG)-induced HK2 cells, and promoted cell fibrosis. We further identified a putative binding site between miR-543 and TSPAN8, which was validated by Dual luciferase reporter assay. The treatment of miR-543 mimic and miR-543 inhibitor could reduce or increase TSPAN8 protein level respectively. We further showed that the overexpression of TSPAN8 could attenuate HG-induced cell injury by reducing fibrosis and increase autophagy. The effects of miR-543 mimic in proliferation, fibrosis, and autophagy were rescued by TSPAN8 overexpression.

**Conclusions:**

Our study indicate that miR-543 mediates high-glucose induced DN via targeting TSPAN8. Interfering miR-543/TSPAN8 axis could serve as potential approach to ameliorate DN.

**Supplementary Information:**

The online version contains supplementary material available at 10.1186/s12882-022-02716-8.

## Introduction

Diabetic nephropathy (DN) is one of the most common and serious complications of diabetes, which can lead to renal failure and fatality [1, 2]. DN has been the main cause of chronic kidney disease, accounting for one-third of cases worldwide [3]. Due to the complex pathogenesis of DN, currently there is no specific treatment for DN. Therefore, understanding the molecular mechanism underlying the pathogenesis of DN could provide insights into the treatments and prevention.

miRNAs are an important class of endogenous non-coding RNAs implicated in a wide range of biological processes and pathological conditions by modulating the expression of the downstream target genes [4]. Recently, a growing body of evidence has suggested the involvement of miRNAs in DN pathogenesis [5]. For example, He et al. demonstrated that miR-320a exacerbates renal dysfunction in DN by sponging MafB and inhibiting the expressions of Nephrin and Gpx3 in podocytes [6]. In contrast, miR-30c plays a protective role against DN by inhibiting the epithelial-to-mesenchymal transition of retinal endothelial cells through targeting Snail1/TGF-β1 signal pathway [7]. miR-543 seems to be implicated in inflammatory responses of a wide range of diseases. Xu et al. showed that exosome-derived miR-543 could reduce lung injury induced by radiation [8]. While in diabetic retinopathy, the inhibition of miR-543 activity could decrease high glucose (HG)-induced cell proliferation, migration, and angiogenesis [9].

TSPAN8 is a member of membrane glycoprotein tetraspanins which are characterized by four highly conserved transmembrane domains. TSPAN8 has been recognized as an important factor in various diseases, such as tumor [10, 11], infectious diseases [12, 13], and immune disorders [14]. The upregulation of TSPAN8 promotes cancer cell stemness [11], and a high expression of TSPAN8 is associated with a poor prognosis in breast cancer [15], colorectal cancer [16], lung adenocarcinoma [17]. However, whether TSPAN8 plays a functional role in DN remains to be elucidated.

In this study, we examined the expression levels of miR-543 and TSPAN8 in DN mice and high glucose (HG) induced HK2 cells. We found that miR-543 was downregulated and TSPAN8 was upregulated in kidney tissues of mice with DN. Moreover, modulating miR-543 could regulate the proliferation, fibrosis, and autophagy in HK2 cells treated with high glucose. TSPAN8 was identified as a target gene of miR-543 and the effect of miR-543 TSPAN8 could be rescued by TSPAN8 overexpression. Our study indicate that miR-543 mediates high-glucose induced DN via targeting TSPAN8.

## Materials and Methods

### Animals

Eight-week-old male db/db mice (*n*=12) on C57BL/KsJ background and control C57BL/KsJ mice (*n*=12) were obtained from NBRI (Nanjing, China). Mice were maintained under a temperature of 21 ± 2 °C and humidity of 55 ± 2%, with a cycle of 12-h light/dark. Mice were allowed to diet and water freely. The db/db mice were commonly used as DN models and previous studies demonstrated that db/db mice could be considered as the early stage of DN at 8 weeks [18–20]. Animal experiments were conducted according to the Guidelines for the Care and Use of Laboratory Animals at The First Affiliated Hospital of Bengbu Medical College and approved by the ethics committee of The First Affiliated Hospital of Bengbu Medical College. Euthanasia of mice was performed using pentobarbital solution (150mg/kg, i.p.).

### Cell culture

Human HK2 cell line purchased from the American Type Culture Collection, ATCC) was cultured in Dulbecco's modified Eagle's medium (DMEM, Gibco, Grand Island, NY) with 15% fetal bovine serum (FBS; Gibco, Grand Island, NY). The medium contained additional 100U/ml penicillin (Gibco, Grand Island, NY) and 100μg/ml streptomycin (Gibco, Grand Island, NY). Cells were cultured in an incubator with 5% CO_2_ at 37°C. For normal growth condition, cells were cultured in medium containing D-glucose at 5.5 mmol/L glucose plus 19.5 mmol/L mannitol. For high glucose (HG) treatment, cells were maintained in medium with 25 mmol/L glucose.

### qRT-PCR

Trizol reagent (Thermo Fisher Scientific) was used to extract RNA from tissues and cells according to the instructions. The extracted total RNA was dissolved in DEPC water and 5 μg of total RNA was used for reverse-transcription into cDNA using RevertAid First Strand cDNA Synthesis Kit (K1622, Thermo Fisher Scientific). The resulted cDNA was diluted to 50 ng/μL and analyzed in a 7500 Real Time PCR System (Applied Biosystems) using SYBR premix EX TAQ II kit (RR820A, Takara). The PCR cycling condition used: 95^o^C 2 mins, 40 cycles of 95^o^C 30 sec, 60^o^C 30 sec and 72^o^C 60 sec. Finally, the 2–∆∆Ct method was used to analyze the relative expression level and GAPDH/U6 was used as the internal reference gene. All primer sequences were synthesized and purchased from Shanghai Sangon Biotechnology Co., Ltd. (Shanghai, China):

miR-543-forward, 5’- CAGTGCTAAAACATTCGCGG-3’; miR-543- reverse,5’-TATGGTTGTTCACGACTCCTTCAC-3’; TSPAN8-forward, 5′- GAGUUUAAAUGCUGCGGUU -3′; TSPAN8- reverse: 5′- AACCGCAGCAUUUAAACUC-3′; GAPDH-forward, 5’-AGAGGCAGGGATGATGTTCTG-3’; GAPDH- reverse, 5’-GACTCATGACCACAGTCCATGC-3’; U6-forward, 5’-ACAGTAGTCTGCACATTGGTTA-3’.

### MiRNA mimics and inhibitor and plasmids

For TSPAN8 overexpression, the cDNA sequence of TSPAN8 was cloned into pcDNA3.1 expression vector. miR-543 mimic and miR-543-inhibitor were purchased from Shanghai GenePharma. For dual luciferase assay, the wide-type (WT) or mutant 3′-untranslated regions (3′-UTRs) of TSPAN8 was cloned into the pmirGLO Dual-Luciferase Vector (Promega, Madison, WI).

Cell transfection was performed using Lipofectamine® 3000 reagent (Thermo Fisher Scientific, L3000001). In 6 well plate, 60% confluent cells were transfected with 100 nM of microRNA mimic or inhibitor or 6 ug of pcDNA3.1- TSPAN8 plasmid according to manufacturer’s instruction. Transfected cells were subjected to subsequent analysis 48 hours post-transfection.

### CCK-8 cell proliferation assay

48 hours after transfection, cells were seeded in to a 96 -well plate at a density of 1500 cell/well and cultured in a humidified cell culture incubator for 0, 24, 48, and 72 hours, respectively. Subsequently, 10 μL CCK8 reaction solution (Dojindo Laboratory, Japan) was added to the cell culture for 3-hour incubation in a humidified cell culture incubator. The light absorption value (OD value) in each condition was captured at 450nm wavelength on a Synergy H1 microplate reader.

### Dual luciferase reporter assay

To demonstrate the functional interaction between miR-543 and TSPAN8 mRNA, the sequence containing the wild type binding site and the sequence with mutated binding site in the 3’UTR of TSPAN8 mRNA were cloned into the PmirGLO vector (Promega). The reporter plasmid and Renilla luciferase (hRlucneo) control plasmid were co-transfected into cells with either miR-543 mimic or miR-NC using Lipofectamine 3000 reagent according to the manufacturer’s instructions. 48 h post transfection, the relative luciferase activities were measured using Dual-Luciferase Reporter Assay Kit (Promega, E1910) on a luminescence microplate reader (Infinite 200 PRO; Tecan). The relative firefly luciferase activity in the reporter plasmid was normalized to that of Renilla luciferase.

### Western blot

Western blotting was conducted as in a previous study [21]. Total protein was extracted from cells using RIPA lysis buffer containing protease inhibitor cocktail (Thermo Fisher Scientific 78429). Cells suspended in RIPA buffer were lysed on ice for 10 mins and lysed cells were centrifuged at 14000 rpm for 10 mins. The supernatant containing total protein lysate was quantified by a BCA Protein assay kit (Beyotime Biotechnology P0009; Shanghai, China). 20 ug of protein was used for SDS-PAGE electrophoresis. Separated protein in SDS_PAGE gel was transferred onto the PVDF membrane (BioRad 1620177, Irvine, CA, USA). After blocking with 5% skimmed milk for 1 hour, the membrane was then incubated with primary antibodies: anti-actin (1:2000, ab8227); fibronectin (1:1000, ab2413), collagen IV (1:1000, ab6586), TSPAN8(1:1000, ab 70007), LC3A/B (1:1000, ab128025), Beclin1 (1:1000, ab62557) and p62 (1:1000, ab91526) overnight. The membrane was washed 3 times with TBST for 5 minutes each. After wash, the membrane was further incubated with HRP-linked secondary antibody (1:3000; Cell signaling #7074, MA, USA) at room temperature for 1 hour. Then the membrane was washed 4 times with TBST and the protein bands were visualized using an enhanced chemiluminescence kit (ECL, Solarbio, Beijing, China) and photographed on a gel imager system (Bio-Rad, Hercules, CA, United States). The densitometry analysis was performed with Image J software (Bethesda, MD, USA).

### Fluorescence in situ hybridization (FISH)

RNAscope kit (Invitrogen, CA, United States) was used to perform RNA FISH according to manufacturer’s instructions. Briefly, the kidney tissues were fixed with 4% paraformaldehyde and embedded in paraffin. 5 μm sections were obtained by a microtome. After deparaffinization and hydration, the tissue sections were hybridized with miR-543 probe with Cy3 fluorescent dye (RiboBio Co. Ltd., Guangzhou, China) at 50°C in hybridization buffer for 3 hours, and the section was mounted onto a slide using the mounting media containing DAPI (Vector Lab, Inc., Burlingame, CA, United States). Fluorescent images were captured by Leica AM6000 microscope.

### Bioinformatics analysis

Starbase (http://starbase.sysu.edu.cn/) online resources was used for bioinformatic prediction of the binding site between miR-543 and TSPAN8 mRNA analysis.

### Statistical analysis

Statistical analyses were performed with SPSS 20.0 software (IBM SPSS, Armonk, NY, USA). The relationship between the expression of miR-543 and TSPAN8 was analyzed by the spearman correlation coefficient test. The statistical difference between two groups was compared using unpaired student’s t tests. Comparisons among multiple groups were analyzed using one-way analysis of variance (ANOVA) with Tukey’s post hoc test for pairwise comparison. Comparisons of data at multiple time points were examined using two-way ANOVA. Data were reported as the mean ± standard deviation (SD). *P* < 0.05, *P* < 0.01 or *P* < 0.001 was considered as statistically significant.

## Results

### The downregulation of miR-543 and upregulation of TSPAN8 in kidney tissue of DN mice

**Fig. 1:**
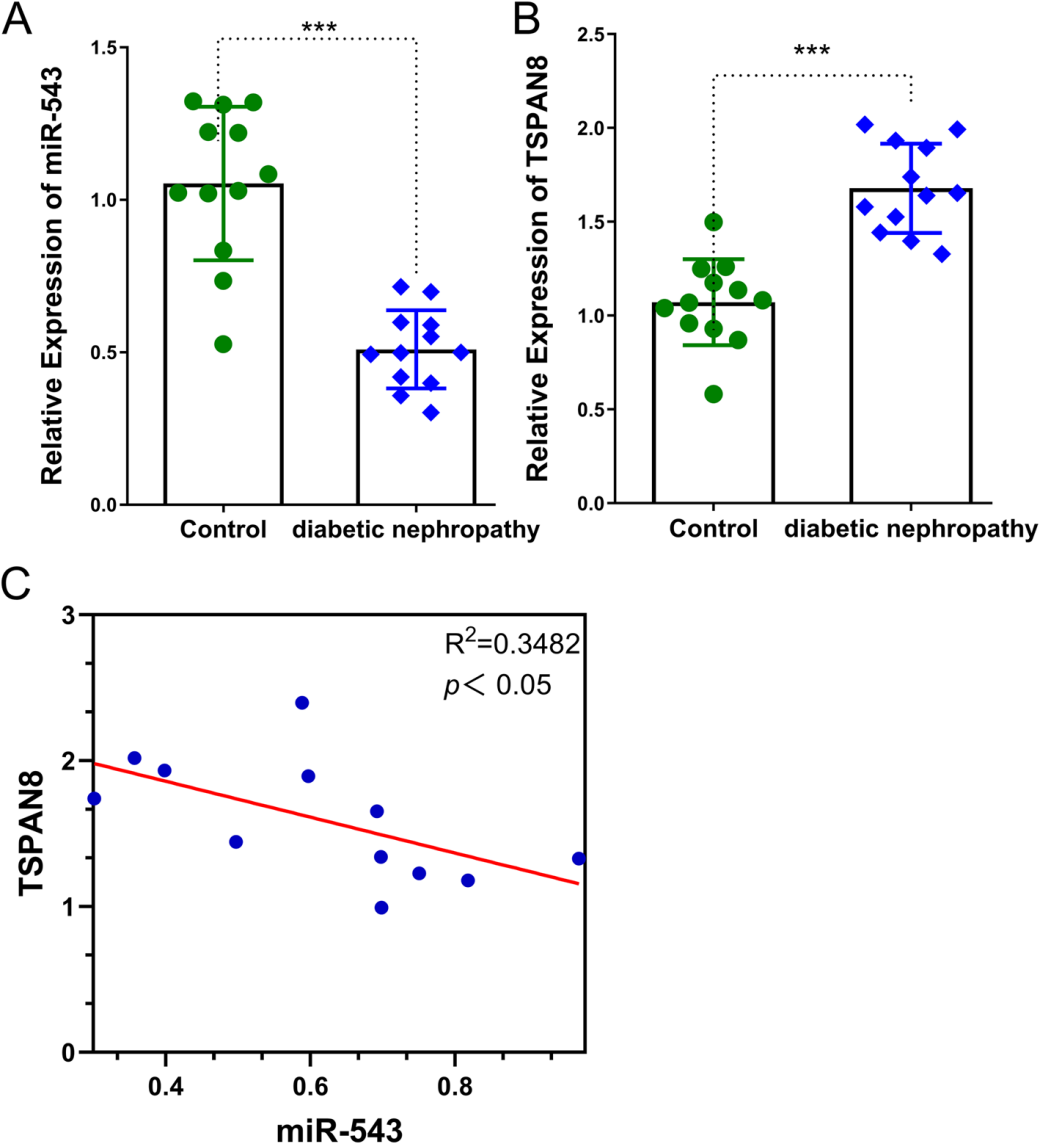
The expression of miR-543 and TSPAN8 in kidney tissue of DN mice. **A** The downregulation of miR-543 in kidney of DN mice (*n*=12) as compared with normal mice (*n*=12). **B** The mRNA expression of TSPAN8 is upregulated in kidney of DN mice (*n*=12) when compared with normal mice (*n*=12). **C** The expression level of miR-54 in DN mice kidney tissues was negatively associated with mRNA level of TSPAN8, as analyzed by the spearman correlation coefficient test. ***p<0.001

We first examined the expression level of miR-543 and TSPAN8 in kidney tissue of DN mice and control mice by qRT-PCR. The results showed that miR-543 was significantly downregulated in DN group compared with the control group (Fig. 1A). We also performed RNA FISH to examine the localization of miR-543 in kidney, and miR-543 seemed to be expressed by different cells in both the cortex and medulla (Supplementary Fig.1) Meanwhile, the expression of TSPAN8 was significantly increased in kidney tissue of DN mice (Fig.1B). Spearman correlation coefficient test demonstrated that miR-543 expression level was negatively correlated with TSPAN8 expression in kidney tissues of DN mice (Fig.1C). These data suggest that both miR-543 and TSPAN8 are dysregulated in DN mouse model.

### miR-543 regulates the proliferation, fibrosis, and autophagy in high-glucose-induced HK2 cell

**Fig. 2:**
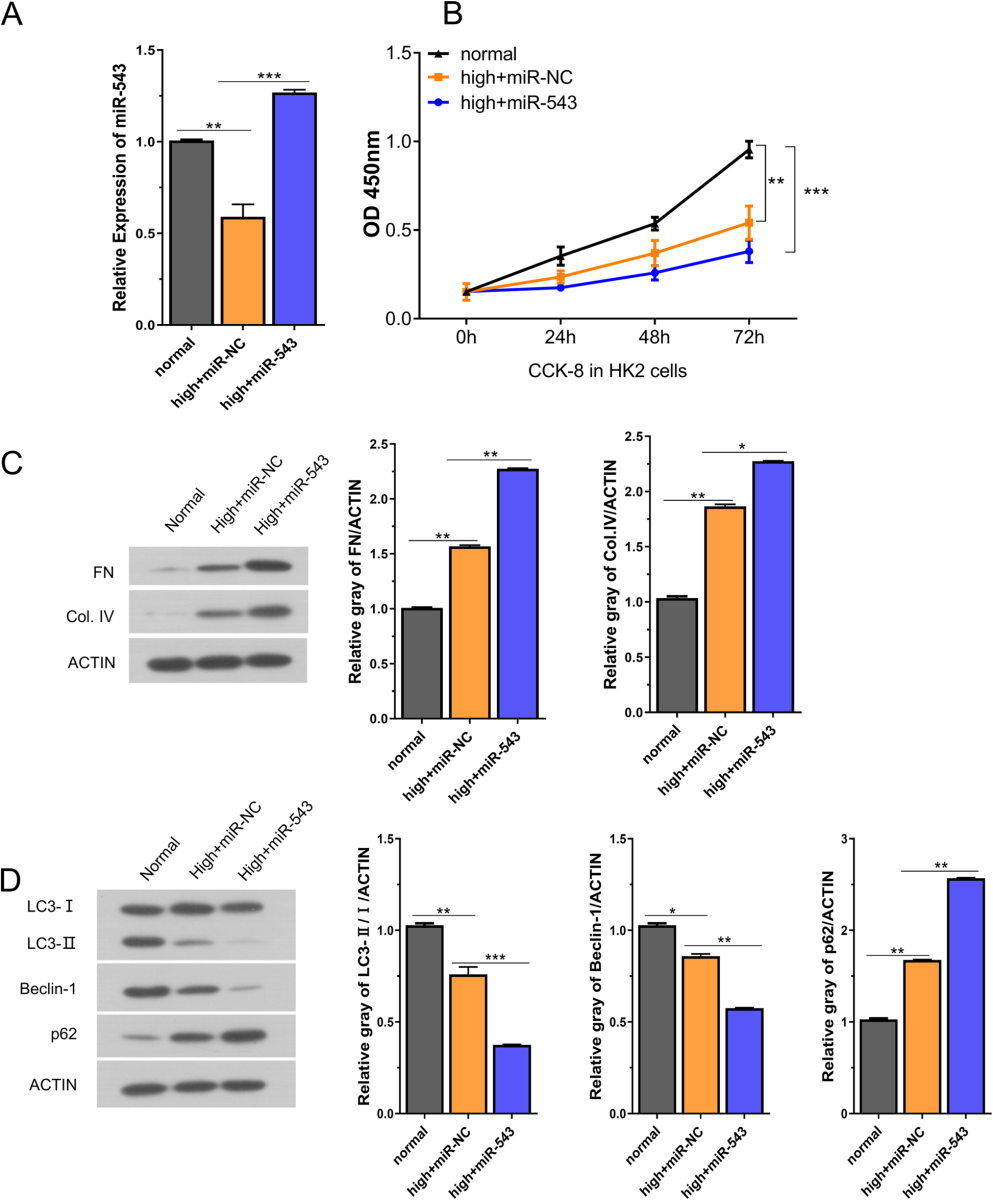
miR-543 regulates proliferation, fibrosis and autophagy in high glucose induced HK2 cell. **A** The expression of miR-543 was detected in different experimental groups (normal group, HG group, HG +miR-543-mimic group) by qRT-PCR. **B** CCK-8 cell proliferation assay in different experimental groups (normal group, HG group, HG+miR-543-mimic group). **C**-**D** The protein levels of fibrosis or autophagy-related proteins were examined in HK2 cells after HG-induction or transfecting with miR-543 mimic. *P < 0.05, **P < 0.01 or ***P < 0.001

To investigate the potential role of miR-543 in DN, we applied miR-543 mimic in high glucose (HG)-induced HK2 cells as a cellular model of DN. HG-treated cells displayed a more elongated and fibroblast-like cell shape, which is reminiscent of fibrotic phenotype (Supplementary Fig.2). The efficiency of miR-543-mimic transfection was confirmed by qRT-PCR (Fig.2A). Compared to miR-NC transfection, miR-543 was significantly increased after miR-543 mimic transfection in HG-induced cells (Fig. 2A). CCK8 cell proliferation assays demonstrated that the HG treatment suppressed cell proliferation, which was further exacerbated by the overexpression of miR-543 (Fig.2B). Moreover, high-glucose treatment increased the expression of collagen IV (Col. IV) and fibronectin (FN), and miR-543 mimic further enhanced their expression (Fig. 2C). We also investigated the change of autophagy-related proteins, which showed that the ratio of LC3-II/LC3-I, Beclin-1 level decreased, but the level of P62 increased upon HG treatment (2D). The overexpression of miR-543 further augmented these changes. These results indicate that high glucose impairs cell proliferation, but promotes fibrosis and autophagy in HK2 cells. miR-543 acts as a promoting factor to further suppress HK-2 cell proliferation and autophagy, but promotes fibrosis in HG-induced HK2 cells.

### miR-543 targets TSPAN8 in HG-induced cells

**Fig. 3:**
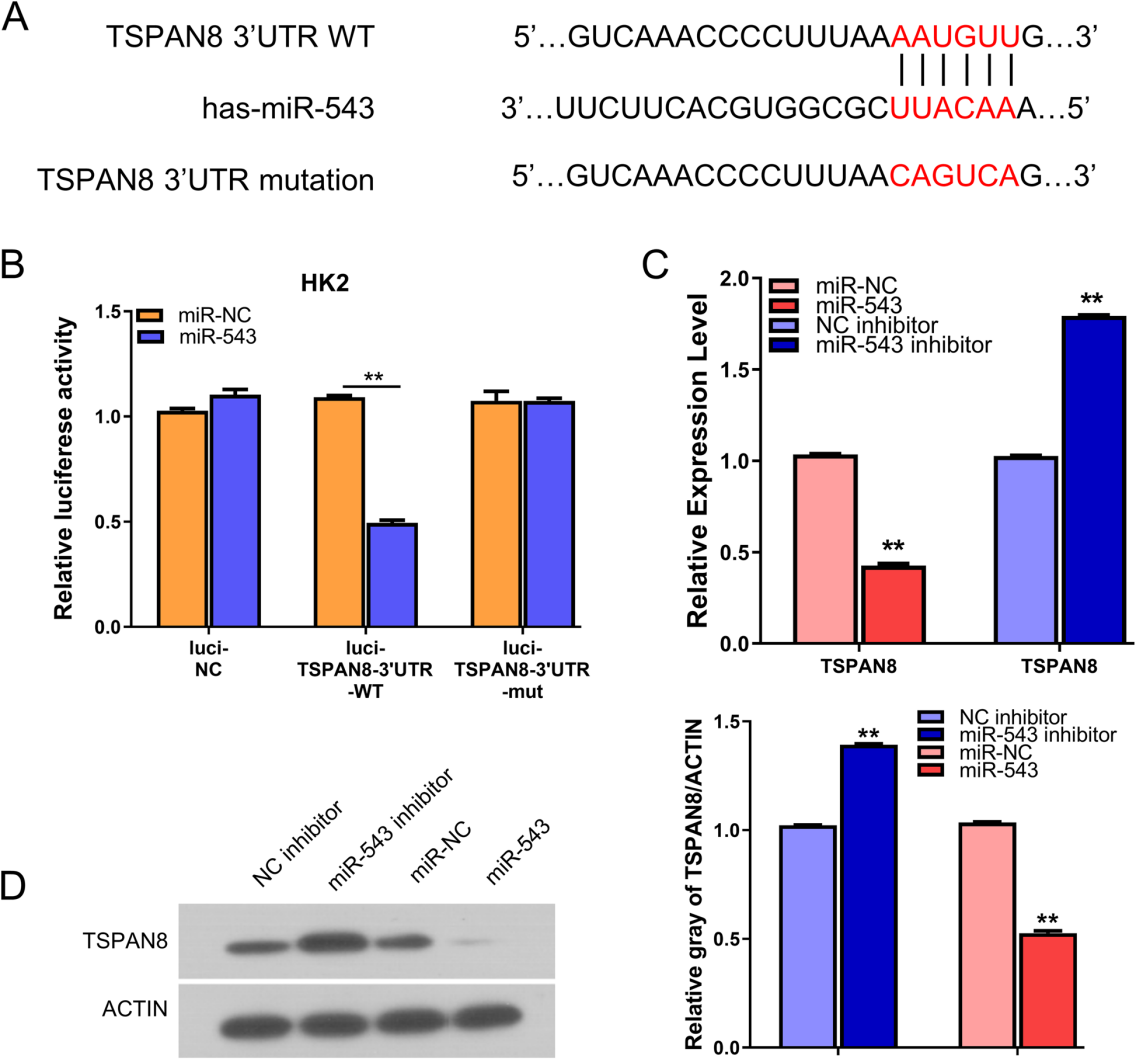
miR-543 negatively regulates TSPAN8 in HK2 cells. **A** The predicted miR-543 binding sites on the 3’UTR of TSPAN8 was shown. **B** Dual luciferase reporter assay was performed using vectors containing WT binding site or mutated binding site between miR-543 and TSPAN8, with the empty vector (Luci-NC) as the negative control. Vectors were co-transfected with miR-NC or miR-543 mimic. **C**-**D** The mRNA and protein levels of TSPAN8 were decreased or increased in HK2 cells after the transfection with miR-543 mimic or miR-543 inhibitor. *P < 0.05, **P < 0.01 or ***P < 0.001

To identify the downstream target gene, the potential binding site of miR-543 was predicted by starbase (http://starbase.sysu.edu.cn/) and there is a putative binding site of miR-543 in the 3’UTR of TSPAN8 (Fig.3A). To confirm their functional interaction, we performed dual luciferase reporter assay with WT and mutated binding site, with the empty vector (luci-NC) as the negative control. We found that the presence of miR-543 mimic significantly suppressed the luciferase activity of WT reporter, which was not observed in the mutated reporter or the negative control vector (Fig.3B). Besides, the mRNA and protein levels of TSPAN8 were reduced after miR-543 mimic transfection, while miR-543 inhibitor increased TSPAN8 expression. (Fig. 3C-D). Together, these data suggest miR-543 negatively regulates TSPAN8 in HG-induced cells.

### TSPAN8 reduces the injury induced by high glucose

**Fig. 4:**
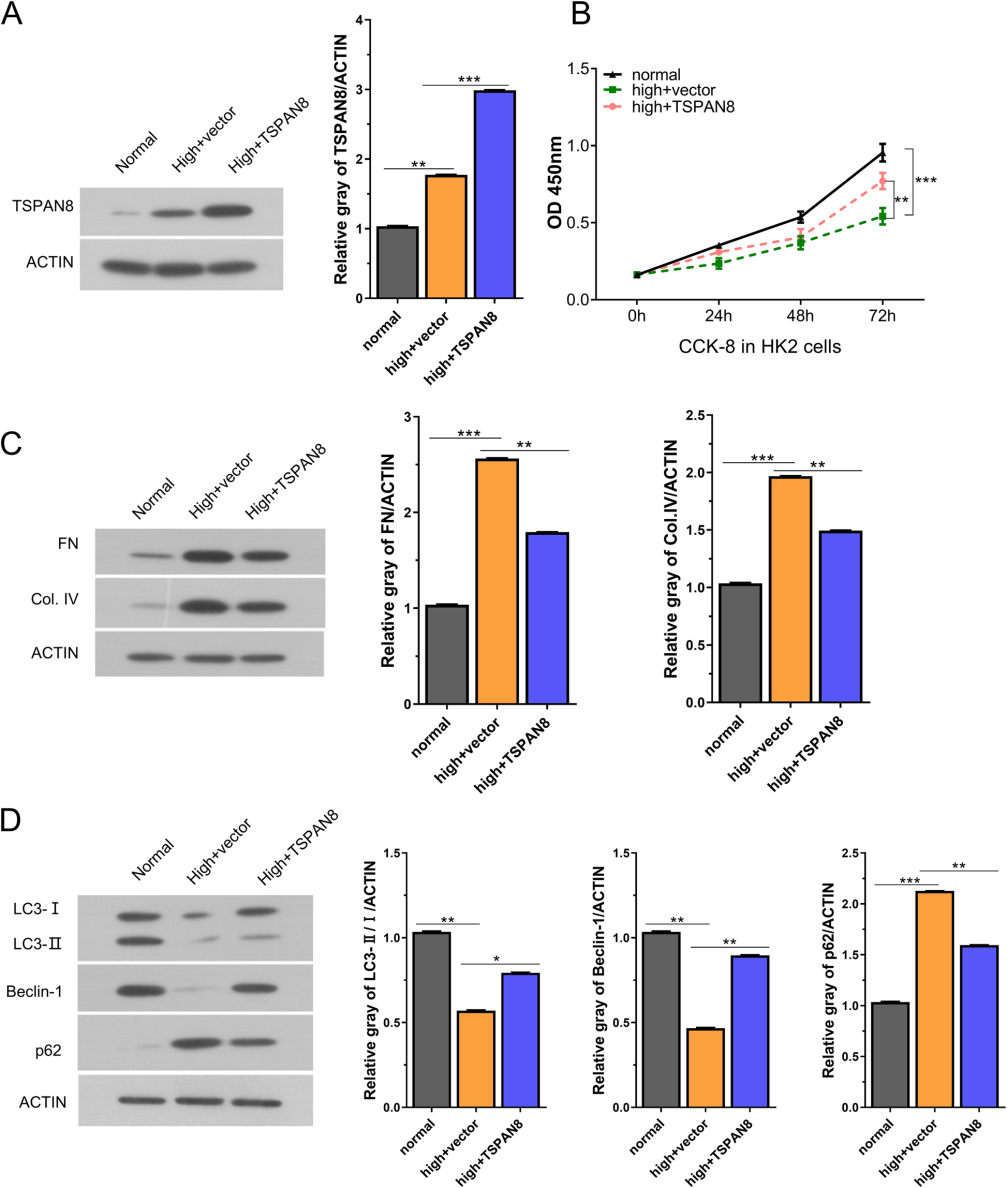
TSPAN8 reduces the injury of HK2 cells caused by high glucose. **A** The expression of TSPAN8 was detected in different groups (normal group, HG group, HG+TSPAN8 group) by Western blot. **B** CCK-8 proliferation assay in different experimental groups (normal group, HG group, HG+TSPAN8 vector group). **C**-**D** The expression of fibrosis or autophagy-related proteins were examined in HK2 cells after HG-induction or the transfection of TSPAN8 overexpression vector. *P < 0.05, **P < 0.01 or ***P < 0.001

To explore the functional role of TSPAN8 in HG-induced cell model, we transfected HK2 cells with TSPAN8 overexpression vector. As shown in Fig.4A, the expression level of TSPAN8 was upregulated in HG-induced HK2 cells after transfecting with TSPAN8 vector. CCK8 assay demonstrated that high glucose could lead to suppression of cell proliferation and TSPAN8 overexpression partially rescued this effect (Fig.4B). In addition, the relative protein levels of LC3-II/LC3-I, Beclin-1 decreased, while fibronectin (FN), collagen type IV (Col. IV), P62 increased in HG-induced HK2 cells upon high glucose induction. TSPAN8 overexpression could partially reverse these effects (Fig.4C-D). These results indicate that TSPAN8 alleviates cell damages induced by HG through suppressing fibrosis and promoting autophagy.

### TSPAN8 alleviates the cellular damages caused by miR-543 overexpression in high glucose-induced cells

**Fig. 5:**
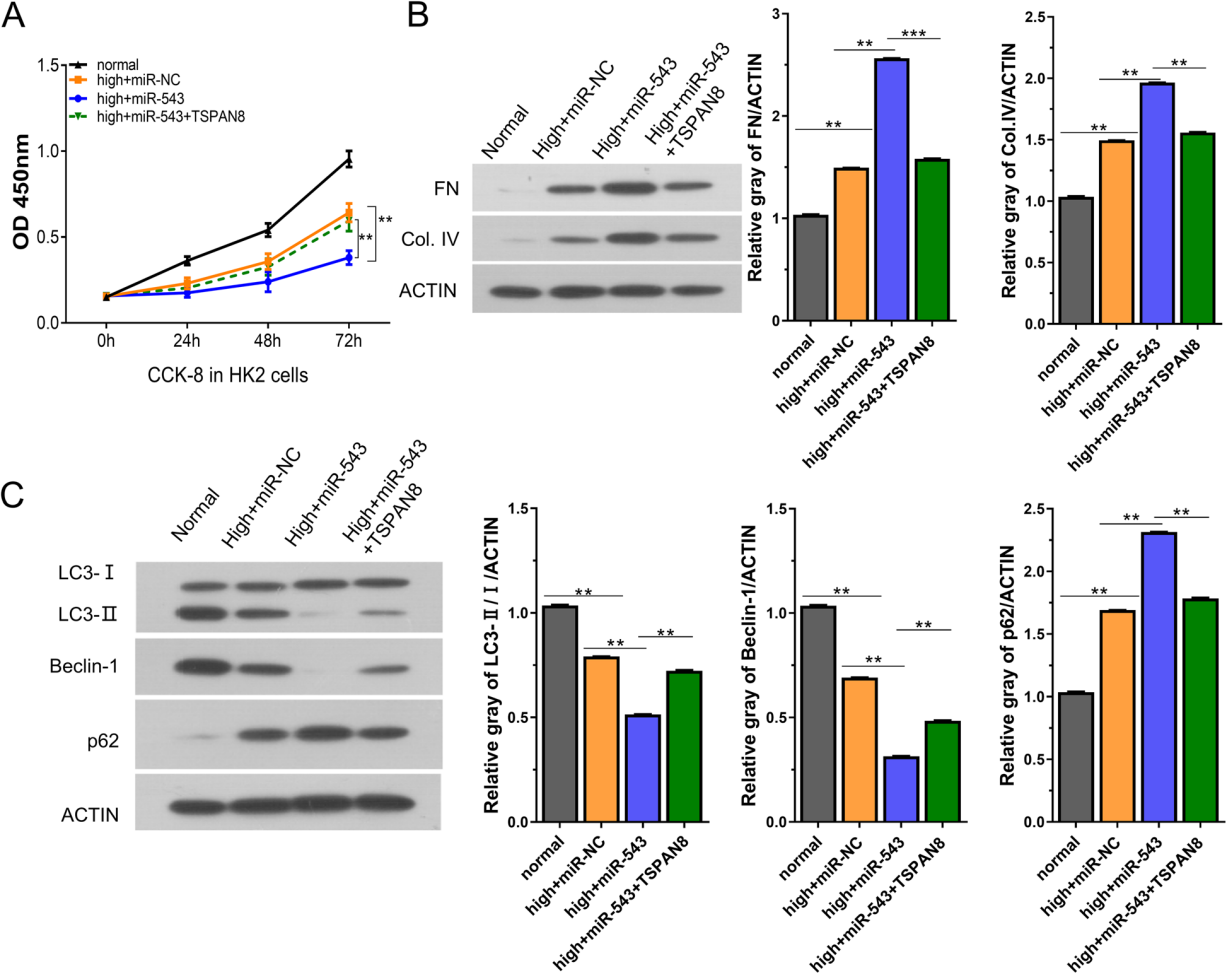
TSPAN8 alleviates the cellular damage caused by miR-543 in high-glucose-induced HK2 cells. **A** CCK-8 proliferation assay in different experimental groups (normal group, HG group, HG +miR-543-mimic group, HG +miR-543-mimic+ TSPAN8 group). **B**-**C** The expression of fibrosis or autophagy related-proteins were detected in different experimental groups (normal group, HG group, HG +miR-543-mimic group, HG +miR-543-mimic+ TSPAN8 group). **P < 0.01 or ***P < 0.001

Given the observation that miR-543 negatively regulates TSPAN8, we further attempted to investigate whether miR-543 mediates high glucose-induced cell damage by targeting TSPAN8. In HG-induced HK2 cells, the cell proliferation was greatly reduced by miR-543-mimic as compared with miR-543-NC and the effect of miR-543 mimic was abrogated by TSPAN8 overexpression (Fig. 5A). The proteins in fibrosis and autophagy were examined in HG-induced HK2 cells with miR-543-mimic and TSPAN8 overexpression. The results showed that miR-543 promoted the decrease of relative ratio of LC3-II/LC3-I and Beclin-1, and increased the levels of FN, Col. IV, and P62, suggesting that miR-543 promotes fibrosis and inhibits autophagy. However, TSPAN8 overexpression could reverse the effects of miR-543 in HG-induced HK2 cells (Fig.5B-C). Together, these data imply that miR-543 mediates high glucose-induced cell damage by targeting TSPAN8.

## Discussion

DN eventually leads to the end-stage renal disease, which is a serious microvascular complications of diabetes mellitus [22]. Recently, many studies have attempted to identify the associated protein changes with DN pathophysiology. For instance, the disorder of inflammatory molecules and pathways like CCL2 and NF-kB pathway are implicated in the progression of DN [23]. However, the potential functions of non-coding RNAs in DN remain largely unknown.

miRNAs have emerged as a class of important non-coding RNAs implicated in the progression of DN. Liu et al. showed that BMP-7 (Bone morphogenetic protein 7) could downregulate the expression level of miR-21, which in turns increase the expression of Smad7 and decrease the expression of Smad3. Consequently, BMP-7 suppresses the epithelial-mesenchymal transition and extracellular matrix deposition to reduce kindey fibrosis in DN [24]. In one study regarding the protective role of Astragaloside on DN, Astragaloside suppresses podocyte apoptosis in rat DN model by targeting miR-378 [25]. Moreover, a previous study has demonstrated that metformin inhibits the senescence of renal tubular epithelial cells in DN by promoting the expression of RNA-bind protein MBNL1 and miR-130a-3p [26]. In this study, we found that miR-543 is downregulated in the kidney tissue of DN mouse model, which is consistent with a previous study in which miR-543 has negative impact on diabetic retinopathy [9].

The functional roles of TSPAN8 have been explored in many cancers. For example, Zhu et al. found that TSPAN8 promotes the stemness and enhances the drug-resistance of breast cancer [27]. In nasopharyngeal carcinoma, colorectal cancer, and melanoma, TSPAN8 also serves as a oncogenic factor to facilitate cell proliferation, invasion, and migration [16, 27, 28]. However, in this study we demonstrated that the TSPAN8 serves as a protective factor for DN. TSPAN8 is a downstream target of miR-543, which shows upregulation in the kidney tissue of DN mouse model. The differential roles of TSPAN8 between cancers and DN may result from the difference in molecular network between cancer and inflammatory disease. Future works are needed to further investigate the mechanisms by which miR-543 is downregulated in DN, and validate the protective effect of TSPAN8 in DN mouse model. In addition, our study only focuses on the HK2 cells derived from proximal tubular cell in kidney. Whether miR-543 and TSPAN8 could exert similar effects on other cells in the glomerulus of kidney (such as podocyte and mesangial cells) need to be further investigated.

## Conclusion

In summary, this study showed the differential expression patterns and functional roles of miR-543 and TSPAN8 in DN mouse model and HG-induced cell model. miR-543 promotes fibrosis, and inhibits cell proliferation and autophagy in HG-induced cell model. TSPAN8 is identified as a target of miR-543, and its overexpression could rescue the effect of miR-543. The protective effect of TSPAN8 in HG-induced cell damage will need to be further validated in DN mouse model.

## Supplementary Information

**Additional file 1: Supplementary Figure S1.** RNA FISH analysis of miR-543 expression in kidney cortex and medulla. Scale bar: 500 μm.

**Additional file 2: Supplementary Figure S2.** Cell morphology of HK-2 cells in normal glucose and high glucose culture condition. High-glucose induced elongated and fibroblast-like cell morphology. Scale bar: 200 μm.

**Additional file 3.**

## Data Availability

The datasets during and/or analyzed during the current study are available from the corresponding author upon reasonable request.
