# Supplementary figures and images for: miR-543 regulates high glucose-induced fibrosis and autophagy in diabetic nephropathy by targeting TSPAN8
Source: BMC Nephrol. 2022 Mar 4;23:89. doi: 10.1186/s12882-022-02716-8 (PMC8895563; doi:10.1186/s12882-022-02716-8)

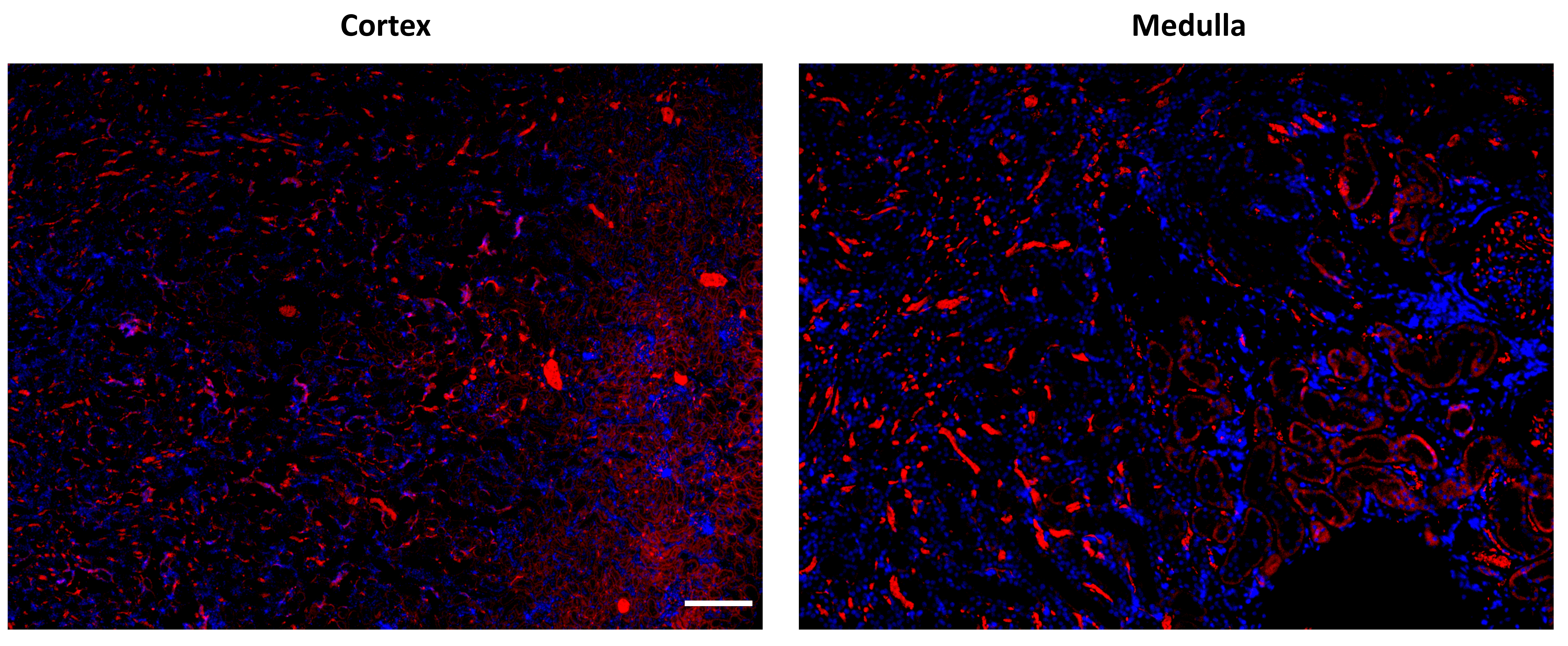

Supplement: Supplementary file 1 — Additional file 1: Supplementary Figure S1. RNA FISH analysis of miR-543 expression in kidney cortex and medulla. Scale bar: 500 μm. [file 12882_2022_2716_MOESM1_ESM.tif]

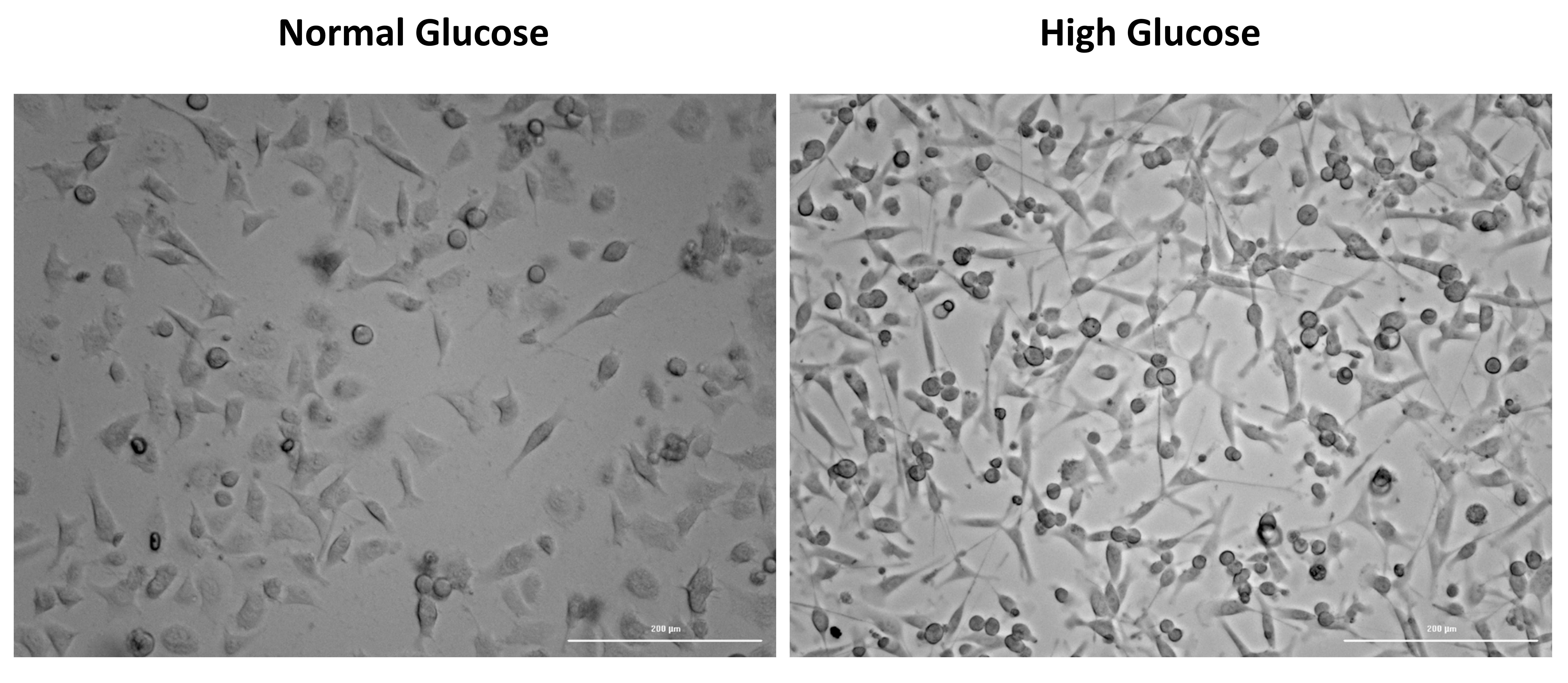

Supplement: Supplementary file 2 — Additional file 2: Supplementary Figure S2. Cell morphology of HK-2 cells in normal glucose and high glucose culture condition. High-glucose induced elongated and fibroblast-like cell morphology. Scale bar: 200 μm. [file 12882_2022_2716_MOESM2_ESM.tif]
