# Supplementary material for: miR-543 regulates high glucose-induced fibrosis and autophagy in diabetic nephropathy by targeting TSPAN8
Source: BMC Nephrol. 2022 Mar 4;23:89. doi: 10.1186/s12882-022-02716-8 (PMC8895563; doi:10.1186/s12882-022-02716-8)
Supplement: Supplementary file 3 — Additional file 3. [file 12882_2022_2716_MOESM3_ESM.pdf]

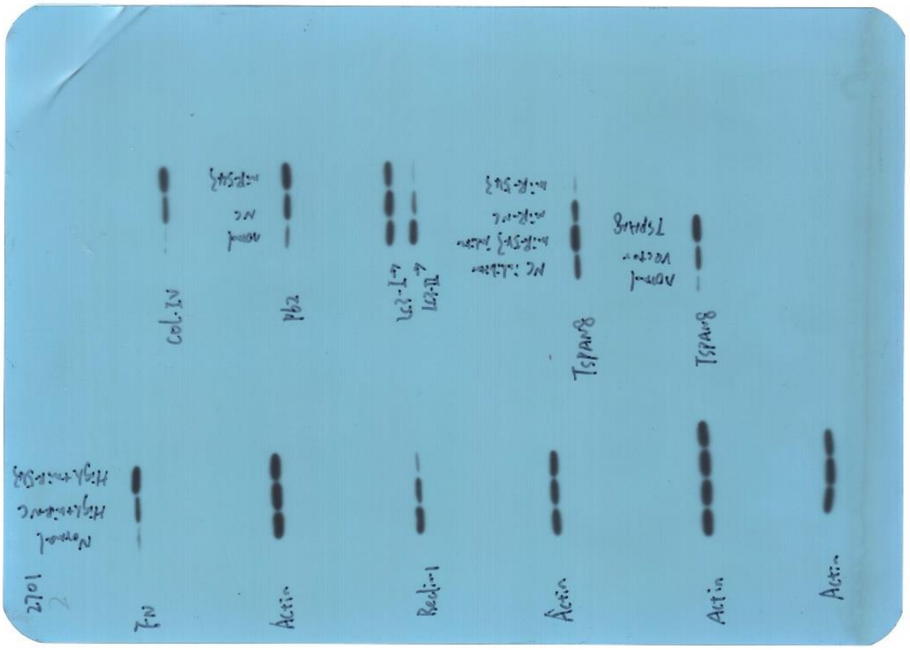

Full scan of Western blot analysis membrane of FN， Col.IV， LC3-I， LC3-II, Beclin-1， p62 and TSPAN8 using actin as the internal control

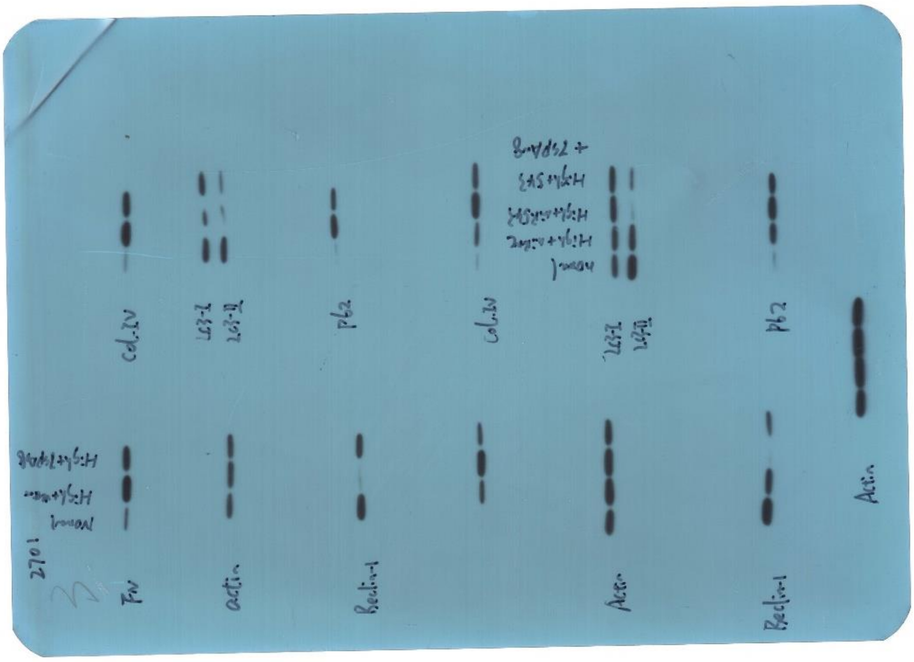

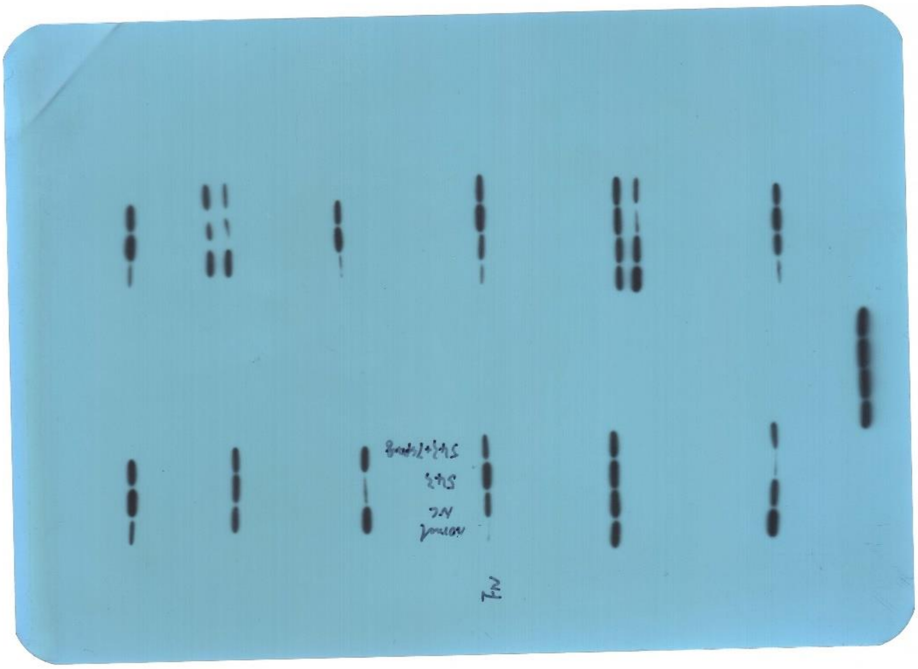

Full scan of Western blot analysis membrane of FN， Col. IV, LC3-I， LC3-II,Beclin-1， p62
